# Using a polygenic score in a family design to understand genetic influences on musicality

**DOI:** 10.1038/s41598-022-18703-w

**Published:** 2022-08-29

**Authors:** Laura W. Wesseldijk, Abdel Abdellaoui, Reyna L. Gordon, 23andMe Research Team, Stella Aslibekyan, Adam Auton, Elizabeth Babalola, Robert K. Bell, Jessica Bielenberg, Katarzyna Bryc, Emily Bullis, Daniella Coker, Gabriel Cuellar Partida, Devika Dhamija, Sayantan Das, Sarah L. Elson, Teresa Filshtein, Kipper Fletez-Brant, Pierre Fontanillas, Will Freyman, Anna Faaborg, Shirin T. Fuller, Pooja M. Gandhi, Karl Heilbron, Barry Hicks, Ethan M. Jewett, Katelyn Kukar, Keng-Han Lin, Maya Lowe, Jey C. McCreight, Matthew H. McIntyre, Steven J. Micheletti, Meghan E. Moreno, Joanna L. Mountain, Priyanka Nandakumar, Elizabeth S. Noblin, Jared O’Connell, Yunru Huang, Aaron A. Petrakovitz, Vanessa Lane, Aaron Petrakovitz, Joanne S. Kim, G. David Poznik, Morgan Schumacher, Anjali J. Shastri, Janie F. Shelton, Jingchunzi Shi, Suyash Shringarpure, Vinh Tran, Joyce Y. Tung, Xin Wang, Wei Wang, Catherine H. Weldon, Peter Wilton, Alejandro Hernandez, Corinna Wong, Christophe Toukam Tchakouté, Fredrik Ullén, Miriam A. Mosing

**Affiliations:** 1 Department of Neuroscience, Karolinska Institutet, Solnavägen 9, 171 77 Stockholm, Sweden; 2 Department of Psychiatry, Amsterdam UMC, University of Amsterdam, Amsterdam, The Netherlands; 3 Melbourne School of Psychological Sciences, Faculty of Medicine, Dentistry, and Health Sciences, University of Melbourne, Melbourne, Australia; 4 Department of Otolaryngology–Head and Neck Surgery, Vanderbilt University Medical Center, Nashville, TN USA; 5 Department of Psychology, Vanderbilt University, Nashville, USA; 6 Vanderbilt Genetics Institute, Vanderbilt University Medical Center, Nashville, TN USA; 7 Department of Cognitive Neuropsychology, Max Planck Institute for Empirical Aesthetics, Frankfurt, Germany; 8 Department of Medical Epidemiology and Biostatistics, Karolinska Institutet, Stockholm, Sweden; 9 23andMe, Inc, Sunnyvale, CA USA

**Keywords:** Genetics, Human behaviour

## Abstract

To further our understanding of the genetics of musicality, we explored associations between a polygenic score for self-reported beat synchronization ability (PGS_rhythm_) and objectively measured rhythm discrimination, as well as other validated music skills and music-related traits. Using family data, we were able to further explore potential pathways of direct genetic, indirect genetic (through passive gene–environment correlation) and confounding effects (such as population structure and assortative mating). In 5648 Swedish twins, we found PGS_rhythm_ to predict not only rhythm discrimination, but also melody and pitch discrimination (betas between 0.11 and 0.16, p < 0.001), as well as other music-related outcomes (*p* < 0.05). In contrast, PGS_rhythm_ was not associated with control phenotypes not directly related to music. Associations did not deteriorate within families (N = 243), implying that indirect genetic or confounding effects did not inflate PGS_rhythm_ effects. A correlation (*r* = 0.05, *p* < 0.001) between musical enrichment of the family childhood environment and individuals' PGS_rhythm_, suggests gene–environment correlation. We conclude that the PGS_rhythm_ captures individuals' general genetic musical propensity, affecting musical behavior more likely direct than through indirect or confounding effects.

## Introduction

Listening to music or engaging actively in music by singing or playing an instrument, is an important part of life for many. Twin research on music-related behavior shows that there is a substantial genetic component to variation in musical engagement, as well as to musical ear, i.e. pitch, melody and rhythm discrimination skills, with heritability estimates ranging between 13 and 86% with an average around 40–50%^1–7^. Twin modelling studies have also shown that there is considerable overlap between the genetic variants underlying such music-related phenotypes (so-called 'genetic pleiotropy'), not only between different specific music skills, but also between music skills and other music-related traits, like music practice or listening to music^3,8,9^. This suggests that there are generalist genes as well as specialist genes underlying variation in music-related traits.

Which particular genes are relevant for music skills is not yet well understood, with the limited existing research largely being based on candidate gene and linkage studies (see Gingras et al.^10^ for review), which are known to suffer from replication problems, and are hampered by small sample size^11^. Recently, a genome-wide association study (GWAS) in a well-powered sample of 606,825 individuals identified sixty-nine genetic variants that influence variation in self-reported beat synchronization ability, assessed with the question: 'Can you clap in time with a musical beat?' Yes/No^12^. The variance explained by the aggregate of these genetic effects (i.e., the SNP-based heritability) was ~ 13–16%. It is not surprising that this estimate is lower than heritability estimates of musical rhythm abilities reported in twin studies (~ 50%), as many common genetic variants with very small effects influence human behavior, requiring extremely large GWAS samples to detect all. In addition, potentially influential rare genetic variants are not yet well captured by current microarray chips^13^. Therefore, up-to-date GWASs capture only a fraction of the expected genetic variation underlying behavioural traits (a phenomenon referred to as 'missing heritability'). Furthermore, although the single item in this GWAS is obviously limited as a measure of musical aptitude, the authors performed extensive validations of this item, showing that it was well-correlated with accuracy in tapping to the beat of musical excerpts and with other related phenotypes such as rhythm perception, a multi-item rhythm questionnaire, and the Goldsmiths test of musical sophistication (see Niarchou et al.^12^ for further details on validation procedures and findings). Most importantly, their GWAS was the first well-powered gene-finding investigation of a music-related trait, and thus allows us to estimate and investigate individuals' genetic rhythmic propensity using a polygenic score (PGS) approach. This approach can provide additional insight into the genetic architecture underlying musical ability, directing future research when disentangling gene–environment interplay underlying musicality and music acquisition.

Over the past decade, post-GWAS analyses were developed to further study genetic variants discovered by GWASs and their associations. In the PGS approach, the estimated effect sizes of genetic variants identified in the discovery sample used in the GWAS, are used as weights in an independent sample, to calculate a score for each individual, representing his or her genetic propensity for the trait under study^14^. It is important to bear in mind that this approach will capture only a fraction of the genetic variation underlying the trait and should not be used to estimate the heritability^13^. We here use the PGS approach to estimate the relationship between the genetics underlying self-reported rhythm ability, as estimated in the beat synchronization ability GWAS (PGS_rhythm_), and objectively measured rhythm discrimination ability, as measured by a well-validated music discrimination test^1^. We will further compare this relationship to the effect the PGS_rhythm_ has on other music skills and phenotypes such as pitch and melody discrimination, motor timing, music practice, music listening, the experience of flow during musical activities and achievement in music. As a control, we investigate associations between PGS_rhythm_ and phenotypes not directly related to music, including general intelligence, sport practice, flow in non-musical activities, and achievement in non-musical domains.

Besides investigating the predictive value and specificity of the PGS_rhythm_, we aim to identify the pathways through which the PGS_rhythm_ affects music skills. Genetic variants can relate to a trait through direct, indirect or confounding effects^15^. A *direct* genetic effect is when genetic variants directly affect the trait^16^. Importantly, this does not mean that the pathway necessarily needs to be simple or 'straight' in mechanistic terms^15^. A different scenario, however, is when a genetic variant affects a trait in an environmentally mediated way, for example, through its effect on parental behavior. This would be an example of an *indirect* genetic effect, i.e., the effect of a genetic variant in one individual on the trait of another individual through the environment^15^. In this case, genes that are shared between parents and children, tend to cause the parents to provide their children with a family environment of a particular type. This chain of events will result in a correlation between a genotype and the family environment, a phenomenon called passive gene–environment correlation (prGE)^17^. For example, parents might have provided a musically enriched family environment, due to their genetic predisposition for music; this environment may facilitate music skill acquisition in their offspring. Individuals with certain genotypes will therefore more often also be exposed to facilitating environments, which can lead to an overestimation of the direct effects of an individual's genetic variants in GWASs and therefore of the PGS. Evidence for this overestimation of effects has been found in previous within-family GWASs that found effects of genetic variants to be much smaller within families^18,19^. There are other *confounding* effects that can inflate the association between a PGS and the trait of interest, such as population stratification and assortative mating. Population stratification occurs when there are differences in allele frequencies between (sub)populations, due to systematic differences in ancestry. This can lead to spurious effects of genetic variants causing inflated estimates of the PGS prediction^20^. Assortative mating refers to a type of non-random mating, namely when partner bonds are established based on similarity on particular phenotypes, like for example, playing a musical instrument. This can also cause an overestimation of the direct effects of genetic variants^21^. In sum, over and above direct genetic effects, indirect genetic effects as well as population stratification and assortative mating can inflate the effect of individual's genetic variants in GWASs and therefore of the PGS on traits of interest^20,21^.

With the use of genetically informative family data, it is possible to disentangle the PGS' direct and combined indirect genetic (referring to prGE) and confounding effects on a trait^15,16,20,22,23^. Dizygotic twins, just as regular siblings, differ at random in the genetic variants they receive from their parents during meiosis, while sharing on average 50% of their genes. Importantly, as the twins of a pair have the same parents, within-pair effects are not biased by population stratification or assortative mating^24^. Furthermore, dizygotic twins share their family environment and have the same age. Therefore, comparing the effect of a PGS on an outcome, between- and within- dizygotic twin pairs, is a way to control for both prGE through the shared family environment, and confounding effects of assortative mating and population stratification. Specifically, if the effect of the PGS on an outcome is larger in a sample of unrelated individuals than within dizygotic twin pairs, this indicates that the effects of the PGS might be inflated by indirect genetic or confounding effects^16,20^. This validated 'within-family PGS' method has recently gained popularity in the field of behavioural genetics. Even so, the idea of direct and indirect genetic effects is not new and has been described and studied with various different designs long before the development of PGSs, for example, in the field of evolutionary quantitative genetics (see for example^25,26^). Lastly, additional analyses can shed light on whether it is more likely that indirect genetic effects or confounding effects are at play. For example, a relationship between a measure of musical enrichment of the family childhood environment and the PGS_rhythm_ would suggest gene–environment correlation (while the absence of a relationship would not exclude its presence). A significant difference from the expected correlation of 0.50 for the PGS_rhythm_ between dizygotic twins could suggest confounding effects such as population structure and assortative mating^15,27^.

In a large genetically informative sample of 5648 Swedish twins, we investigated the association between the PGS of self-reported beat synchronization ability (PGS_rhythm_), and a well-validated measure of rhythm ability, other music skills (pitch and melody discrimination), motor timing and music-related phenotypes such as music practice and listening behaviour, musical flow experiences and lifetime achievement in music. As control phenotypes, we included general intelligence, sports practice, global and work-related flow experiences as well as dance-, science- and writing-related achievements. We further explore the potential pathways of direct, indirect and confounding effects of the PGS_rhythm_ on music skills using within-family analyses, and examine the possibility of gene–environment correlation and assortative mating.

## Methods

### Participants

The Study of Twin Adults: Genes and Environment (STAGE) is a cohort of the Swedish Twin Registry (STR) which includes approximately 32,000 adult twins born between 1959 and 1985^28,29^. In 2012 and 2013, 11,543 twins from this cohort completed a web survey on, among other things, musical engagement, musical aptitude, motor timing (finger tapping), flow proneness and achievements. More details on these phenotypes, their distributions and heritability estimates can be found in earlier published studies using this sample see^1–3,8,30–32^. In 2019 and 2020, individuals from the STR, who provided saliva samples between 2006 and 2008, were genotyped. After quality control, genotype data were available for 8,343 individuals from the STAGE cohort, of which 5648 had also completed the web survey in 2012/2013. Informed consent was obtained from all participants. The study and analyses of biomarkers were approved by the Regional Ethics Review Board in Stockholm (Dnr 2011/570-31/5, Dnr 2018/960-31/2, Dnr 2019-05879). The computations and data handling were enabled by resources provided by the Swedish National Infrastructure for Computing (SNIC) at Uppsala partially, funded by the Swedish Research Council through grant agreement no. 2018-05973. All research methods were performed in accordance with relevant guidelines and regulations.

### Measures

#### Start age of playing music

Participants answered whether they ever played a musical instrument (including singing) and, if so, at what age they started.

#### Amount of music practice

Total lifetime music practice of the participants was estimated based on questions about start and ending age of playing music and on the participant's indication of the number of hours per week they practiced (in 10 categories ranging from 0 h, via more than 6–9 h, to more than 40 h) during four age intervals (ages 0–5, 6–11, 12–17 and 18 years until time of measurement). From these answers and information on the numbers of years they played music, we calculated an estimate of total lifetime amount of music practice.

#### Hours of music listening

Individuals were asked how many hours per week they listened to music on average since they were 18 years old until the measurement.

#### Musical aptitude

Musical aptitude was measured using the Swedish Musical Discrimination Test (SMDT)^1^. The SMDT was administered online and includes three subtests: a pitch, melody and rhythm discrimination test. The pitch (27 items), melody (18 items) and rhythm (18 items) discrimination scores were standardized before calculating a mean overall musical aptitude score. The SMDT has been shown to have good reliability and internal consistency (reliabilities and alphas between 0.79 and 0.89) and inter-correlations between the discrimination scales ranged between 0.27 and 0.41^1^.

#### Motor timing

Motor timing (or finger tapping) was measured using the isochronous serial interval production (ISIP) paradigm^33^. In the ISIP task, participants tap in synchrony with a regular, auditory metronome, followed by a phase where the participant has to continue to tap self-paced without a metronome. After an initial training trial, the participants completed six experimental trials with varying inter-onset intervals (in the order of 524, 819, 655, 1024, 655, and 524 ms). The motor timing score was calculated as the mean coefficient of variation during the self-paced phase across the six trials. We reversed the score so that a higher score indicated a more accurate performance. For detailed information on the task and scoring see Mosing, et al.^30^.

#### Creative achievement, including achievement in music

Achievement was measured with an adapted and translated version of the Creative Achievement Questionnaire (CAQ)^2,34–36^. This instrument is a self-report measure of achievement in the visual, music, dance, writing, invention, science, and theater domains using a seven-point scale. As an example, items to rate achievement in music range from 1 ‘I am not engaged in music at all’ via 4 ‘I have played or sung, or my music has been played in public concerts in my home town, but I have not been paid for this’ to 7 ‘I am professionally active as a musician and have been reviewed/featured in national or international media and/or have received an award for my musical activities’. Apart from music, we included dance, which also might be associated with rhythm ability, as well as writing and science as control domains.

#### Musical enrichment of family childhood environment

The level of musical enrichment of childhood environment was estimated as previously by Wesseldijk et al.^2^, i.e. using the first principal component derived from the following family environment measures: (1) the number of records available in the family home, (2) how many individuals in the twin’s environment played an instrument, (3) how often they visited concerts and (4) whether or not they were offered music education before the age of 12.

#### General intelligence

Intelligence was measured with the Wiener Matrizen Test (WMT), a visual matrix test similar to Raven’s standard progressive matrices^37^. The test is 25-min long and consists of 24 multiple-choice items, which are scored either one (correct) or zero (incorrect or missing response). A sum of the 24 item scores was used as a measure of the participant's psychometric intelligence.

#### Flow, including flow proneness in the musical domain

Flow proneness was measured with the Swedish Flow Proneness Questionnaire (SFPQ)^38^. The SFPQ is a self-report measure of the frequency of psychological flow experiences in the domains work, leisure, maintenance and music. All four sub-scales consisted of seven items each rated on a 5-point Likert scale ('never' to 'every or almost every day'). A global flow proneness was calculated as the mean score of flow proneness in work, leisure and maintenance. We included besides flow proneness in the musical domain, global flow proneness and flow proneness in the domains of leisure and work for comparison. See Ullén et al.^38^ for a more detailed description of the scale.

### Genetic data processing

A total of 8442 twins from the STAGE cohort were genotyped using the Illumina Infinium assay (chip GSAMD-24v1-0_20011747_A1). Before standardized quality control, samples were excluded in case of discrepancies in observed sex and relatedness (*N* = 86). The remaining samples were processed using the Ricopoli pipeline for quality control^39^, which led to the exclusion of 13 more samples. Quality control of the single nucleotide polymorphisms (SNPs) led to the exclusion of 581 SNPs, resulting in 491,839 included genotyped SNPs. These SNPs were imputed using the Haplotype Reference Consortium panel (HRC1.1)^40^. PLINK 2.0 sets were generated, and a light post-imputation quality filter was applied, leaving 9,141,508 markers. This resulted in a total sample size of 8343 twin individuals (2554 complete twin pairs including imputed monozygotic twin pairs). Of these, 5648 individuals (2303 monozygotic, 1581 same-sex dizygotic, 1663 opposite-sex dizygotic and 101 individuals of which zygosity was unknown) provided information on music variables. Of which 1184 same-sex and opposite-sex dizygotic complete twin pairs provided genetic information, and 243 dizygotic and opposite-sex dizygotic complete twin pairs provided both genetic and music information.

A principal component analysis (PCA) was performed in the full study sample (including 28 samples identified as non-European ancestral outliers as they remained in the genotype files as they passed all other filters) to generate ancestry covariates. PLINK 1.9 and 2.0 were used to extract the first 20 principal components (PCs) based on common independent genotyped markers (minor allele frequency (MAF) > 0.05, pairwise R^2^ < 0.1). The first 10 PCs were included as covariates in further PGS analyses, to control for potential confounding by population stratification, i.e., when there are systematic differences in allele frequencies between subpopulations^41^.

### Polygenic score (PGS_rhythm_) calculation

To create polygenic scores (PGSs) for the 8343 individuals, we extracted a restricted set of common, well-imputed 1,265,094 HapMap 3 SNPs from their genotype data^42,43^. We generated PGSs based on summary statistics from the large GWAS on self-reported beat synchronization ability in N = 606,825 individuals of European ancestry (Niarchou et al.^12^ participating in research with the personal genetics company, 23andMe, Inc. Participants in the GWAS had provided informed consent and participated in the research via internet, under a protocol approved by the external AAHRPP-accredited IRB, Ethical & Independent Review Services (E&I Review). Polygenic scores are a weighted sum of each individual’s trait-associated alleles at each SNP multiplied by that SNP’s estimated effect size as detected by a GWAS^14^. The effect sizes were first re-estimated using the summary-data based on the best linear unbiased prediction (SBLUP) approach^44,45^. This approach computes effect sizes with best linear predictor properties that account for linkage disequilibrium between SNPs. As a reference sample for the linkage disequilibrium, a random sample of 11,064 unrelated individuals was extracted from a set of 1,246,531 HapMap 3 SNPs that passed quality control in the UK Biobank sample^46^. PGSs were generated, based on these re-estimated effect sizes, for the 8,343 samples that passed quality control, using PLINK 1.9. Scores were then imputed for monozygotic co-twins (*N* = 1350) and merged with the music data and the top 10 of the 20 obtained PCs from the PCA.

### Statistical analyses

All analyses were performed in STATA. All variables, with the exception of sex and age and the PCs, were standardized.

#### Predictions of the PGS_rhythm_

To investigate the relationship between the genetic variants underlying self-reported beat synchronization ability (PGS_rhythm_) and objectively measured rhythmic ability, we performed a linear regression analysis, with the PGS_rhythm_, the top 10 PCs to control for ancestry structures, sex and age as independent variables and the rhythm discrimination score as the dependent variable. Further linear regression analyses were performed to explore the effect of the PGS_rhythm_ on other music-related outcomes, namely pitch and melody discrimination, general musical aptitude, motor timing, amount of music practice, achievement in music, flow proneness in the musical domain, and start age of playing music. In addition, to investigate how specific PGS_rhythm_ predictions were to the music domain, we investigated associations between the genetic variants underlying rhythm discrimination and control phenotypes, i.e. general intelligence, amount of sport practice, flow proneness in non-musical domains and achievements in dance, writing, and science. To estimate an effect size of the PGS_rhythm_ (variance explained R^2^) for each dependent variable, we subtracted the total R^2^ of the model without the PGS_rhythm_ from the total R^2^ of the full model (with PGS_rhythm_). All regression analyses included sex, age and the top 10 PCs as covariates. We always corrected for relatedness in the twin sample by using the robust standard error estimator for clustered observations^47,48^. All analyses were first performed including imputed genetic data for monozygotic twins (*N* = 5648) and then repeated with only one of the monozygotic twin members included (*N* = 4787).

#### Pathways of direct, indirect and confounding effects of the PGS_rhythm_

To explore the potential pathways of direct, indirect and confounding effects of the PGS_rhythm_ on rhythm discrimination, we utilized the family structure (twins) of the sample. We applied a recently developed and validated random intercept mixed-effects model on the dizygotic twin data^16^. In this model, two fixed effects are fitted to partition the total effect of the PGS_rhythm_ on the outcome into a between-family (${\beta }_{B})$ and within-family effect $({\beta }_{W}$) (see Equation adapted from Selzam et al.^16^)

$${Y}_{ij}= {\propto }_{0}+{\beta }_{W}\left({PGS}_{ij}-{\overline{PGS}}_{j}\right)+ {\beta }_{B}{\overline{PGS}}_{j}+ {\gamma }_{j}+ {\varepsilon }_{ij}$$

*Y* denotes the outcome and PGS the polygenic score, *i* = {1,2} corresponds to the individual twins that are clustered within family *j,* and $\overline{PGS}$ refers to the mean PGS value in family *j*. Therefore, the between-family effect represents the expected change in outcome *Y* given a one unit change in the family PGS average, while the within-effect represents the expected change in outcome given a one unit change in the difference between the individual PGS and the family average PGS. If the between-effect of the PGS on the outcome is significantly larger than the within-effect, indirect genetic or confounding effects may be inflating the effect of the PGS^16,20^. If the within- and between-effect are similar in size this provides evidence for direct effects of the measured genetic variants. Including both the between- and within-family effect in the same model, causes adjustment and independence of the two individual estimates, and offers the possibility to compare the confidence intervals for significance. See Selzam et al.^16^ for more details about the model. Only complete dizygotic twin pairs were included in these analyses (*N* = 243). The analysis included sex and 10 PCs as covariates. We repeated this analyses with musical aptitude as an outcome and we repeated the analysis only in same-sex dizygotic twin pairs (*N* = 128).

#### Additional testing for gene–environment correlation and potential confounding

To further investigate possible indirect or confounding effects of the PGS_rhythm_ on outcomes, namely gene–environment correlation and population structure confounding or assortative mating, we performed two additional analyses. We calculated the correlation between an index of the level of musical enrichment of the family childhood environment and the PGS_rhythm_. A significant correlation would indicate that an individual’s genetic make-up (in terms of rhythmic ability) is related to their (perceived) childhood environment, which could be interpreted as further support for gene–environment correlation. We also tested for the potential presence of confounding, such as assortative mating or other types of population structure confounding, by calculating a correlation between the dizygotic twins for the PGS_rhythm_. If 0.50 is not included in the confidence interval of the correlation, this would indicate a significant difference from the expected genetic correlation of 0.50 for the PGS_rhythm_ between siblings.

## Results

### Predictions of the PGS_rhythm_

**Figure 1:**
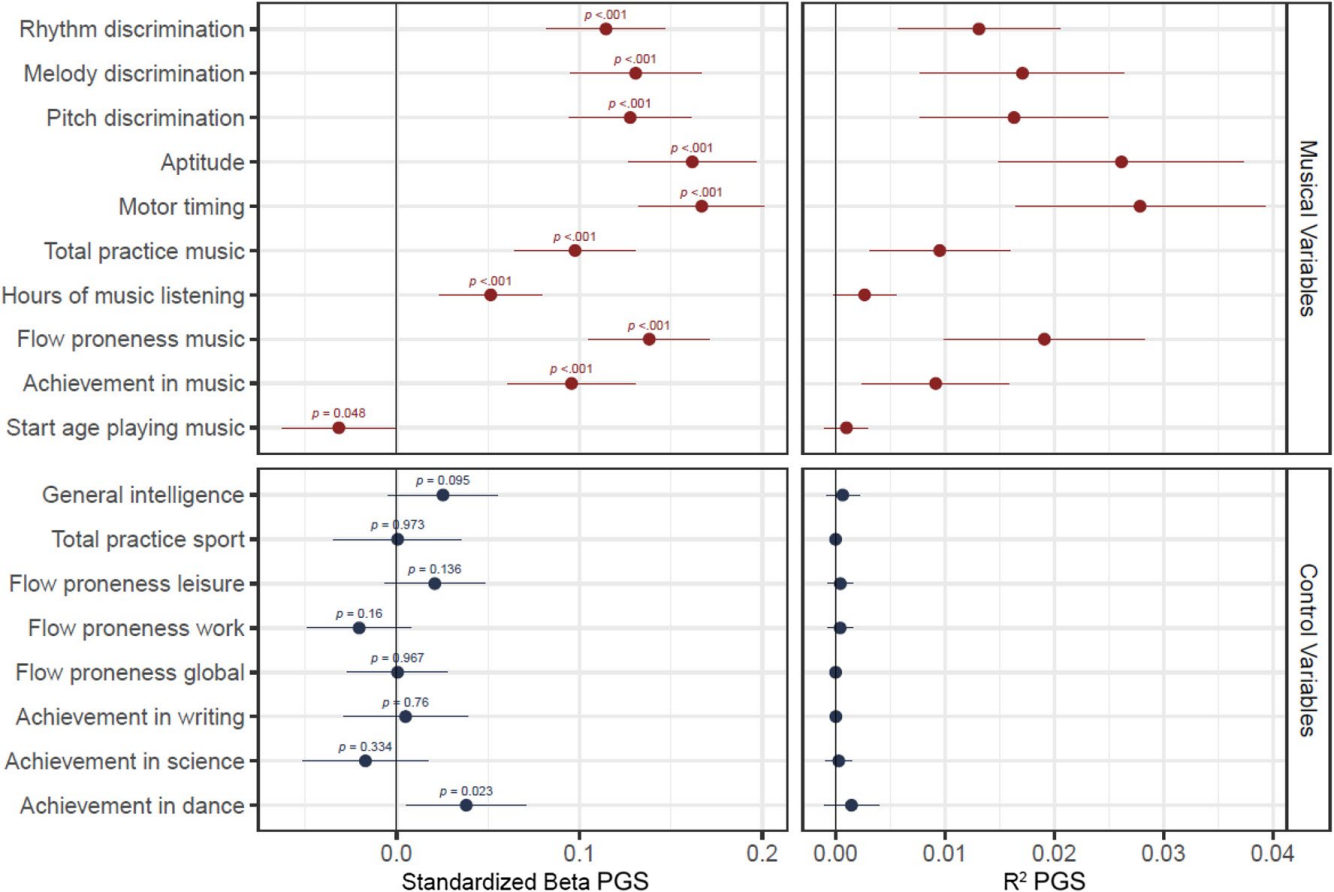
Effects of the PGS_rhythm_ on music-related variables and control phenotypes. The association between PGS_rhythm_ and start age playing music indicates that a higher PGS_rhythm_ is associated with starting to play music at a younger age.

The polygenic score for self-reported beat synchronization ability significantly predicted rhythm discrimination skill (*β* = 0.11, p < 0.001, R^2^ = 0.01). Notably, effects for melody discrimination (*β* = 0.13, p < 0.001, R^2^ = 0.02), pitch discrimination (*β* = 0.13, p < 0.001, R^2^ = 0.02), and the overall musical aptitude score (*β* = 0.16, p < 0.001, R^2^ = 0.03) were also significant, and nominally higher than for rhythm discrimination. The PGS_rhythm_ also significantly predicted motor timing (*β* = 0.17, p < 0.001, R^2^ = 0.03) and all other music related outcomes (Fig. 1, upper part).

The PGS_rhythm_ did not predict individual differences in general intelligence, total sport practice, and flow proneness or achievements in domains other than music (Fig. 1, lower part). A small but significant effect was seen on achievement in dance (*β* = 0.04, p = 0.02, R^2^ = 0.001). Analyses in the sample excluding imputed monozygotic twin data were nearly identical (see Supplementary Table 1).

### Pathways of direct, indirect and confounding effects of the PGS_rhythm_

Within- and between-family PGS_rhythm_ prediction estimates and their confidence intervals from the within-family analyses are displayed in Table 1. Within-family effects were certainly not lower—in fact, nominally higher—than between-family effects, but the partly overlapping confidence intervals indicate no significant difference between within- and between-family effects. This suggests that indirect genetic or confounding effects did not significantly inflate the effect of the PGS_rhythm_. Estimating within- and between-family PGS_rhythm_ effects only within same-sex twins did not change these results.

**Table 1:** Within- and between-family estimates of the effect of the PGS_rhythm_ on levels of rhythm discrimination and musical aptitude in a sample of 243 complete dizygotic twin pairs.

	Beta	Std. error	P-value	CI lower 95%	CI upper 95%
**Rhythm discrimination**					
Between	0.04	0.04	0.28	− 0.03	0.12
Within	0.15	0.07	0.04	0.02	0.28
**Aptitude**					
Between	0.06	0.03	0.28	− 0.04	0.16
Within	0.19	0.09	0.04	0.01	0.37

### Additional testing for gene–environment correlation and potential confounding

There was a small but significant correlation between the level of musical enrichment of family childhood environment and individuals' PGS_rhythm_ (*r* = 0.05, p < 0.001, CI 0.02–0.08), supporting the presence of gene–environment correlation. In addition, the PGS_rhythm_ correlation between dizygotic twin members was slightly higher than expected at 0.54 (*p* < 0.001, CI 0.50–0.58). Note that this trend was only borderline significant, with the confidence interval including 0.50.

## Discussion

### Validity and specificity of the polygenic score for self-reported rhythm ability

We investigated the predictive value of a polygenic risk score (PGS_rhythm_) for self-rated beat synchronization ability on objective rhythm ability, other musical skills and music-related outcomes, as well as on a set of control outcomes, not directly related to music. Our results support the validity of PGS_rhythm_, in that we indeed found an association between the genetic variants underlying self-reported beat synchronization ability and objectively measured rhythm discrimination, aligning with phenotypic associations reported in Niarchou et al.^12^. Moreover, PGS_rhythm_ did not only predict rhythm skill but tapped broadly into music-related phenotypes. Interestingly, PGS_rhythm_ thus predicted melody discrimination, pitch discrimination and the overall musical aptitude score as well as it predicted rhythm discrimination, if not better. Further, as expected, the PGS_rhythm_ most strongly predicted individual's motor timing skills, converging with neuroscience findings on the role of the motor system in beat synchronization^49^. In addition, associations with PGS_rhythm_ were also significant for other music-related traits, such as total amount of music practice, music listening behavior, achievement in music, flow proneness in the music domain, as well as achievement in dance, a domain in which one would expect enhanced sensitivity to rhythm, and to music more generally.

Furthermore, discriminative validity of the PGS_rhythm_ for music-related traits was demonstrated by the fact that we found no associations with traits of relevance for domain-general expertise (intelligence, general flow proneness) and creativity (in writing and science); nor with engagement in another control domain (sports). Overall, the data thus indicate that the PGS_rhythm_ to a large part captures generalist genes underlying the music domain, rather than specialist genes only relevant to rhythmic ability. In other words, the underlying genetic variants essentially appear to tap into a complex of music-related traits that include overall musicality as well as a tendency to enjoy and engage with music, rather than only specifically reflecting a genetic predisposition to rhythmic propensity.

As already touched upon in the introduction, the discovery GWAS on which the PGS_rhythm_ was based, identified genetic variants that are associated with whether individuals think they can clap in time with a musical beat or not. Although carefully validated, this binary self-report of beat synchronization ability is a very simplistic and minimal phenotype, as it is a broad 1-item yes/no question that is also likely to capture variation in general musical ability (e.g. whether an individual thinks that they are musical in general) and we cannot rule out some degree of rater bias in the GWAS sample. Niarchou and co-authors (2021) demonstrated that their derived polygenic score for beat synchronization was related to broad musician status in a health care context (OR = 1.33); however, no other musical phenotypes were available in their cohort to compare and determine specificity of the PRS^12^. It would be expected, and has been shown for other traits like depression^50^, that a risk score derived from a GWAS on a more specific, reliable, and objective measure of music skill would show higher discriminant validity for different music abilities (i.e. capture more specialist genes). Indeed, as mentioned in the introduction, using twin modelling in the same sample, we have previously found evidence for both specific genetic influences (specialist genes) on rhythm, pitch, and melody discrimination^51^, and on perceptual and motor aspects of rhythmic skill^31^, as well as factors shared between the different musical traits (generalist genes). This lends further support for the notion that the PGS_rhythm_ captures more music generalist genes, rather than the genes specific to rhythmic ability.

The present findings are in line with recent empirical findings and models of expertise, which propose that a high level of domain-specific skill is associated with clusters of relevant traits across different modalities of individual differences, including abilities, as well as e.g. personality, physical trait, and interests^52^. Conceivably, the PGS_rhythm_ thus taps into genes that pleiotropically influence a whole complex of music-related traits, which facilitate expertise acquisition within music and music-related domains, and include abilities as well motivational factors relevant to musical expertise (music flow, music-practicing behavior).

### Pathways of the polygenic score for self-reported rhythm ability on music skills

We further explored potential pathways through which the PGS_rhythm_ affects music skills—direct, indirect and confounding effects—using a within-family design. If a PGS between unrelated individuals has a larger impact on a trait than the PGS has within dizygotic twin pairs, indirect genetic or confounding effects may be inflating the PGS effect^16,20^. As mentioned in the introduction, among indirect genetic effects are PGS_rhythm_ effects that are mediated by the family environment due to passive GE correlation (prGE). However, we did not find evidence for that indirect (that is prGE) or confounding effects (e.g., population stratification or assortative mating) inflated of the association between PGS_rhythm_ and musical aptitude and rhythm discrimination; specifically, the effects of the PGS_rhythm_ on music skills were not significantly lower within dizygotic twin pairs. Previous studies have found that for several traits related to socio-economic status (mainly educational attainment), indirect genetic effects and passive gene–environment correlations do inflate the effects of PGSs, whereas this appears not to be the case for traits unrelated to educational attainment^16,18,19^. It could be that rhythm discrimination skills are less associated with educational attainment than other music-related behavior, like musical engagement.

### Gene–environment correlation and potential confounding

We found the level of musical enrichment of the family childhood environment to be associated with individuals' PGS_rhythm._ This provides evidence for a role of gene–environment correlation for music skills, as individuals' genotypes correlate with their family environment. Although our within-family analyses suggest that prGE may not be relevant for the effect of the PGS_rhythm_ on music skill, this correlation could reflect reactive or active gene–environment correlations. Reactive gene–environment correlation is when different conditions are evoked from the environment depending on genetically influenced phenotypes of the individual (e.g. musicality), and active gene–environment correlation will occur when genetic factors influence an individual’s tendency to actively search out and create specific environmental conditions. Neither of these forms of gene–environment correlation can be disentangled with a within-family design.

We found a borderline significant trend for potential confounding effects, such as assortative mating or residual population structure effects, with a PGS_rhythm_ correlation of exactly 0.50 between dizygotic twin members being the lower limit of the confidence interval (r = 0.54, CI 0.50–0.58). Assortative mating has been established for traits like height, body mass index^53^, educational attainment^54^ and psychiatric problems^55,56^, but has also been indicated for personality traits and values^57^. Preferences for similarities with a mate, including music and art preferences has also been reported^58^. Although it seems plausible that assortative mating could occur based on levels of musicality or a tendency to enjoy and engage with music, Okbay et al.^27^ recently showed for educational attainment that an inflated mate-pair PGS correlation most likely reflects other residual population structure effects or possibly more complex mating patterns rather than assortative mating. As the within-family analyses did not indicate an inflation due to prGE or confounding effects (which would include assortative mating) of PGS_rhythm_ on music skills and the PGS_rhythm_ has a relatively low prediction accuracy compared to the PGS of education attainment (which is based on a much larger discovery GWAS), the observed inflation of PGS_rhythm_ correlation between dizygotic twins does not lend strong support for the presence of assortative mating effects.

### Strengths, limitations and conclusion

This is the first study to investigate the validity and specificity of the self-reported rhythm genotype in a large target sample of individuals that had completed well-validated music discrimination tests and a rich array of questionnaires about musical engagement. Moreover, it is the first study that investigates the genetic basis of music skills using a polygenic score approach, which is a substantial improvement to candidate gene approaches. However, despite using a powerful discovery GWAS and a large genetically informative sample, variance explained by the PGS_rhythm_ for music outcomes is small. This is in line with PGS studies on other phenotypes. When the sample size of the GWASs increase, prediction accuracy of PGSs is expected to also increase with an expected *r*^2^* of h*^2^_*snp*_/(1 + (*M/N* × *h*^2^_*snp*_)), where *h*^2^_*snp*_ is the heritability captured by the GWAS, *M* is the number of independent genetic variants and *N* is the discovery sample size^13^. Therefore, even larger sample sizes and better measures of (objective) music skills will surely be needed to increase the predictive value and accuracy of a musicality genotype in the future. A methodological challenge is that this requires harmonization of music related phenotypes across multiple genetically informative samples. Further, the availability of family data allowed us to explore potential pathways of direct genetic, indirect genetic and confounding effects of the genetic variants associated with self-reported rhythm. It is important to note that one should be careful when trying to isolate and interpret 'pure' direct genetic effects with sibling analyses, as measurement error due to genetic nurture effects can attenuate the associations^59^. Additionally, it is yet unknown whether our results apply to rare genetic variants, since the GWAS and our polygenic scores are based on common genetic variants only. Lastly, findings from the within-family analyses are based on only complete dizygotic twin pairs, which resulted in a much smaller sample of 243 twin pairs. Lack of power could therefore cause a failure to detect a difference between within- and between-family effects and therefore we may have missed possible indirect and confounding effects of the PGS_rhythm._

To conclude, we investigated the validity and specificity of a self-reported rhythm genotype using the PGS approach. Our findings showed that the PGS_rhythm_ predicts overall musicality as well as a tendency to enjoy and engage in music including achievement in dance. The PGS_rhythm_ affects music skills and music-related behavior more likely through direct genetic effects rather than through genetic effects mediated by the family environment or confounding effects. Further, we found evidence for gene–environment correlation for music-related traits and the potential presence of assortative mating for self-reported beat synchronization ability. In this study, we showed that a polygenic score based on a GWAS that had been conducted on a minimalistic and broad music-related item can serve as a valid proxy for individuals' general genetic musical propensity, tapping into the genetic architecture underlying the broader music domain. Overall, our findings show that this PGS_rhtyhm_ can reliably be used to further disentangle gene–environment interplay underlying the broader musical trait complex, but it also lends hope that, as more genetically informative samples with minimal musical phenotyping become available, researchers in this emerging field may be able to join forces for a GWA-meta-analysis to identify additional generalist music genes and enhance our understanding of the genetic architecture of musicality.

## Supplementary Information

Supplementary Table 1.

## Data Availability

The datasets generated during the current study cannot be made publically as registry data and data from the company 23andme were used. Individuals are able to apply online at the Swedish Twin Registry to access the twin data. Our analyses code is available at https://osf.io/shqft/.
