# Supplementary material for: Using a polygenic score in a family design to understand genetic influences on musicality
Source: Sci Rep. 2022 Aug 29;12:14658. doi: 10.1038/s41598-022-18703-w (PMC9424203; doi:10.1038/s41598-022-18703-w)
Supplement: Supplementary file 1 — Supplementary Table 1. [file 41598_2022_18703_MOESM1_ESM.docx]

**Supplementary material**

Supplementary Table 1. PGS_rhythm_ predictions in the sample without imputed MZ twins (*N* = 4,787). Significant beta's are bold.

|  | N | Beta | SE | P-value | R^2^ PGS |
| --- | --- | --- | --- | --- | --- |
| Rhythm discrimination | 3,242 | **0.11** | 0.02 | <.001 | 0.0126 |
| Melody discrimination | 3,196 | **0.14** | 0.02 | <.001 | 0.0199 |
| Pitch discrimination | 3,164 | **0.12** | 0.02 | <.001 | 0.0137 |
| Aptitude | 3,162 | **0.17** | 0.02 | <.001 | 0.0261 |
| Motor timing | 3,200 | **0.17** | 0.02 | <.001 | 0.0269 |
| Total practice music | 3,478 | **0.10** | 0.02 | <.001 | 0.0091 |
| Hours of music listening | 4,783 | **0.05** | 0.02 | 0.001 | 0.0025 |
| Flow proneness music | 3,296 | **0.15** | 0.02 | <.001 | 0.0202 |
| Achievement in music | 3,202 | **0.10** | 0.02 | <.001 | 0.0089 |
| Start age playing music | 3,478 | **-0.03** | 0.02 | 0.044 | 0.0012 |
| General intelligence | 3,880 | 0.03 | 0.02 | 0.094 | 0.0007 |
| Total practice sport | 2,929 | 0.01 | 0.02 | 0.522 | 0.0001 |
| Flow proneness leisure | 4,505 | 0.03 | 0.02 | 0.083 | 0.0007 |
| Flow proneness work | 4,319 | -0.02 | 0.02 | 0.185 | 0.0004 |
| Flow proneness global | 4,592 | 0.00 | 0.01 | 0.874 | 0.0000 |
| Achievement in writing | 3,202 | 0.01 | 0.02 | 0.542 | 0.0002 |
| Achievement in science | 3,202 | -0.02 | 0.02 | 0.383 | 0.0003 |
| Achievement in dance | 3,202 | **0.06** | 0.02 | 0.001 | 0.0034 |
